# Linear Growth through 12 Years is Weakly but Consistently Associated with Language and Math Achievement Scores at Age 12 Years in 4 Low- or Middle-Income Countries

**DOI:** 10.1093/jn/nxy191

**Published:** 2018-11-01

**Authors:** Alysse J Kowalski, Andreas Georgiadis, Jere R Behrman, Benjamin T Crookston, Lia C H Fernald, Aryeh D Stein

**Affiliations:** 1 Nutrition and Health Sciences Doctoral Program, Laney Graduate School, Emory University, Atlanta, GA; 2 Hubert Department of Global Health, Rollins School of Public Health, Emory University, Atlanta, GA; 3 Brunel Business School, Brunel University London, Uxbridge, United Kingdom; 4 Economics, Sociology and Population Studies Center, University of Pennsylvania, Philadelphia, PA; 5 Department of Public Health, Brigham Young University, Provo, UT; 6 School of Public Health, University of California Berkeley, Berkeley, CA

**Keywords:** height-for-age *z* score, growth, cognitive achievement, Young Lives

## Abstract

**Background:**

Whether linear growth through age 12 y is associated with language and math achievement at age 12 y remains unclear.

**Objective:**

Our objective was to investigate associations of linear growth through age 12 y with reading skill, receptive vocabulary, and mathematics performance at age 12 y in 4 low- or middle-income countries (LMICs).

**Methods:**

We analyzed data from the Young Lives Younger Cohort study in Ethiopia (*n* = 1275), India (*n* = 1350), Peru (*n* = 1402), and Vietnam (*n* = 1594). Age 1, 5, 8, and 12 y height-for-age *z* scores (HAZ) were calculated. Language and math achievement at age 12 y was assessed with the use of country-specific adaptations of the Peabody Picture Vocabulary Test, the Early Grades Reading Assessment, and a mathematics test; all test scores were standardized by age within country. We used path analysis to examine associations of HAZ with achievement scores. Twelve models were examined at each age (3 tests across 4 countries).

**Results:**

Mean HAZ in each country was <–1.00 at all ages. Overall, linear growth through age 12 y was associated with 0.4–3.4% of the variance in achievement scores. HAZ at 1 y was positively and significantly associated with the test score in 11 of the 12 models. This association was significantly mediated through HAZ at 5, 8, and 12 y in 9 of the models. HAZ at 5, 8, and 12 y was positively and significantly associated with test scores in 8, 8, and 6 models, respectively. These associations were mediated through HAZ at older ages in 6 of the HAZ at 5-y models and in 6 of the HAZ at 8-y models.

**Conclusion:**

Child relative linear growth between ages 1 and 12 y was weakly but consistently associated with language and math achievement at age 12 y in 4 LMICs.

## Introduction

Linear growth in the first 1000 d (conception through the second birthday) is widely used as a proxy measure for child development. Several studies have shown that growth faltering in the first 1000 d is associated with poorer performance on assessments of cognition concurrently (1–4), in later childhood (3, 5–7), adolescence (7, 8), and adulthood (9). Similarly, lower height-for-age *z* scores (HAZs) were associated with poorer schooling attainment in an analysis of data from 5 birth cohorts in Brazil, Guatemala, India, the Philippines, and South Africa (10). Children exposed to a nutritional supplement during the first 1000 d were taller, performed better on cognitive development assessments, had increased schooling attainment, and the men had increased earnings, relative to children from control communities (9–12).

Whether linear growth after the first 1000 d continues to be associated with measures of cognition remains unclear. In a meta-analysis of cross-sectional associations of linear growth with cognitive development, growth after age 2 y was associated with higher cognitive development scores, although the effect size for the adjusted pooled estimate was less than half of the effect size before age 2 y (13). It is worth noting that the estimates for children ≤2 y old were derived from 3 studies and the estimates for children >2 y old were derived from 7 studies (13). In a cross-sectional study, the association of HAZ and language achievement at age 6–8 y held in a study population with low levels of stunting and after controlling for anemia and lead toxicity, factors also associated with language achievement (14).

Longitudinal studies suggest that improvements in height with age or recovery from stunting are associated with improved performance on cognitive development assessments (5, 7, 15–18). In a longitudinal study in the Philippines, children's height was measured at 6 mo, 24 mo, 8 y, and 11 y, and a summary measure of cognitive ability at age 11 y was created from measures of nonverbal intelligence, reading comprehension, and mathematics (15, 18). HAZ at 6 mo, change in HAZ from 6–24 mo, and change in HAZ from 2 to 11 y were each positively and significantly associated with cognitive ability at 11 y (15). In studies examining recovery from stunting, children who were stunted in infancy and recovered by age 5 y performed as poorly as children who remained stunted in South Africa (5). In contrast, in the Philippines, children who were stunted at age 2 y but had recovered by age 11 y had improved cognitive achievement relative to children who remained stunted (7).

Young Lives (YL) is a longitudinal study of childhood poverty in 4 low- and middle-income countries (LMICs)—Ethiopia, India, Peru, and Vietnam (19)—and several studies have examined the associations between growth and language and mathematics achievement with the use of data from the YL cohorts (3, 16, 20–22). In an analysis of the Younger Cohort in Peru, a greater HAZ at 6–18 mo and greater HAZ at 4.5–6 y were both positively associated with language and quantitative reasoning ability at age 4.5–6 y (3), and children who were stunted at age 1 y but who recovered by age 4.5–6 y performed as well as children who were never stunted, and significantly better than children who remained stunted, on tests of language and quantitative reasoning (23). In analyses of the Younger Cohorts, children who grew faster than their peers at all ages performed better on language and mathematics assessments at age 8 y (16), and children who had recovered from stunting at age 8 y performed better on language and mathematics assessments than children who were persistently stunted, although they performed worse than children who were never stunted (20). Lastly, in an analysis of the YL Older Cohort, children who recovered from stunting from age 8 y to 15 y had improved language and mathematics scores at age 15 y compared with children who remained stunted (21).

In this paper, we use data from the YL Younger Cohort to examine the associations of child relative linear growth [relative to the age-specific WHO medians (24, 25)] through age 12 y, with language and math scores at age 12 y. Although positive associations between relative linear growth from ages 1–8 y and language and math achievement scores were already found, it is worthwhile examining these associations at age 12 y, when children have greater independence and exposure to other factors such as years of schooling and school quality, which could influence language and mathematics achievement.

## Methods

### 

#### Study population

We analyzed data from YL, a longitudinal study of childhood poverty in Ethiopia, India (the states of Andhra Pradesh and Telangana), Peru, and Vietnam (19). Briefly, countries were selected to provide a wide range of cultural, political, geographic, and social contexts and children were sampled from 20 sentinel sites in each country, with poor sites oversampled. The YL study contains an Older Cohort and a Younger Cohort—we analyzed data from the Younger Cohort and did not include the Older Cohort because the latter were recruited at age 8 y and their growth in early life is not known. The Younger Cohort consisted of ∼2000 children enrolled in each country when they were age 6–18 mo and has undergone 4 waves of data collection that have been made available to the public to date (2002; 2006–2007; 2010–2011; and 2013–2014). As of the fourth wave of data collection, <5% of the children in each country had been lost to follow-up. The YL protocol was reviewed by the Central University Ethics Committee of the University of Oxford and country-specific ethics committees. Collective consent was obtained within communities and informed consent was obtained from children and caregivers as appropriate.

#### Nutritional status measures

Supine length (age 1 y) and height (ages 5, 8, and 12 y) were measured with the use of standardized length boards and stadiometers (26). Length-for-age *z* scores and HAZ were calculated through the use of the 2006 WHO standards for children younger than 5 y (24) and the 2007 WHO reference for children ≥5 y (25). As described elsewhere (20), we adjusted the Round 1 data to account for the pattern of decline in HAZ with age within the sample by adding the difference between a child's measured HAZ at Round 1 and the mean country-specific HAZ for all children within 1 mo of the child's age to the mean country-specific HAZ for children aged 11–13 mo. This adjustment is preferable to adding age as a covariate in the model because it does not assume a linear relation between HAZ and age. Similar adjustments were not made for data from subsequent data rounds because at those ages, in contrast to age 1 y, there was no association between HAZ and age at measurement.

#### Language and math achievement measures

Language achievement was assessed at age 12 y via the Peabody Picture Vocabulary Test (PPVT) and the Early Grade Reading Assessment (EGRA). Mathematics achievement was assessed via a mathematics achievement test (referred to as Math henceforth). The PPVT is a widely used test of receptive vocabulary that includes items consisting of a stimulus word and a set of 4 pictures, and requires the child to select the picture that best represents the meaning of the stimulus word presented orally by the examiner. In Peru, the 125-item Spanish version of the PPVT was used, whereas for the other 3 countries the 204-item PPVT-III was adapted and standardized in each country. The EGRA is designed to measure basic, foundational skills for literacy acquisition in early grades (27). Three subtests of the EGRA were adapted to assess word familiarity, reading comprehension, and listening comprehension (28). The Math test included 29 items on counting, number discrimination, knowledge of numbers, and basic operations with numbers.

Extensive analysis of the psychometric characteristics of the tests in Round 3 indicated high reliability and validity of test items (28). Test administration procedures and training were standardized across fieldworkers and the tests were administered in children's homes in spaces that were as quiet as possible. In Ethiopia and Peru, the tests were administered in multiple languages to allow children to respond in the language in which they felt most comfortable. In India, the PPVT was administered in Telugu and the Math test was administered with the use of a test booklet with questions in Telugu and English, whereas the EGRA was administered in both Telugu and English. We used the child's highest standardized EGRA score in the analysis; for 49.8% of children this was their Telugu score. In Vietnam, the tests were administered in Vietnamese.

We normalized each child's PPVT, EGRA, and Math scores, representing a child's deviation from the mean score of peers their same age (in completed months) within their country. Owing to small numbers in the tails of the age distribution, the youngest 50 and the oldest 50 children in each country were each collapsed into a single category.

#### Control variables

For all analyses, we controlled for a set of potential confounders at the child, parental, household, and community levels, as well as test characteristics. These covariates were selected a priori based on their likelihood to affect test performance, and we used the same set of covariates that were used in the Round 3 analysis for consistency (16). We adjusted for child sex and parental sociodemographic characteristics, including: caregiver age when the child was 12 y, caregiver ethnicity, caregiver and paternal highest school grade attained, and maternal height (97.1%, 99.5%, 99.0%, and 99.1% of caregivers are biological mothers in Ethiopia, India, Peru, and Vietnam, respectively). Household characteristics included the YL child's birth order and the logarithm of total monthly per capita household consumption expenditure at age 12 y. We controlled for the fixed effects for sentinel site at enrollment and an indicator variable for moving out of the sentinel site in subsequent rounds. We also controlled for the language of test administration and whether the test was administered in the child's native language.

#### Analytic sample

We identified outlier HAZ observations as absolute HAZ values >5.0 or an absolute difference in HAZ between any 2 rounds >4.0. We set individual outlier observations to missing. For 22 children with outlier change scores we could not determine the erroneous data point; these children's data were excluded from the analysis.

For our descriptive results we included children who were age 6–18 mo at enrollment and provided 4 valid HAZ measures. For our main results we further restricted our sample to include children with covariate information and achievement test scores. Because the number of children completing each achievement measure varied, we allowed the sample sizes for the different achievement models to vary, to maximize the sample sizes (**Supplemental Figure 1**).

#### Statistical models

**FIGURE 1:**
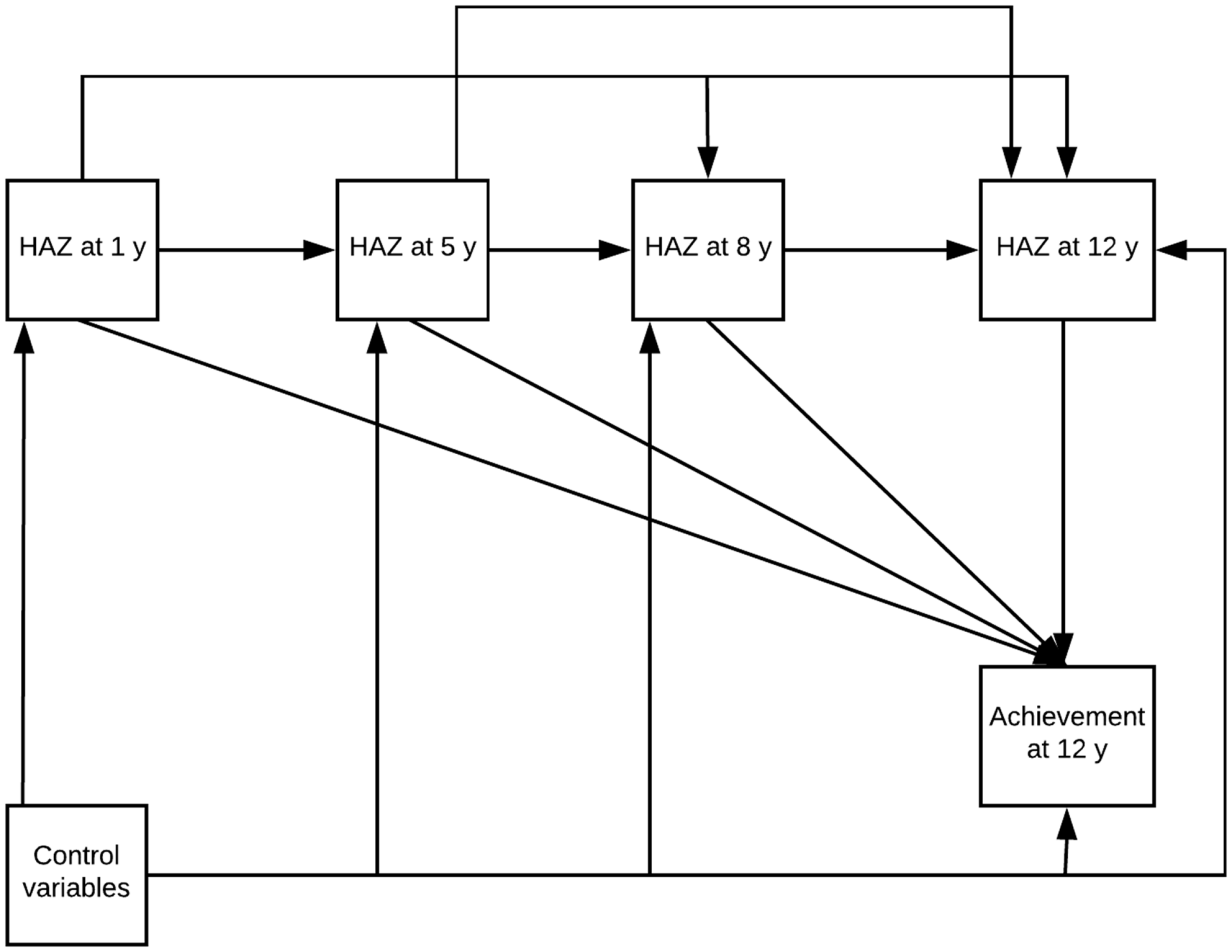
Path diagram for the associations between HAZ at ages 1, 5, 8, and 12 y and language and math achievement at age 12 y. HAZ, height-for-age *z* score.

We used path analysis to examine the associations of child HAZ at 1, 5, 8, and 12 y with PPVT, EGRA, and Math scores at age 12 y (**Figure 1**). Path models describe how the variance between HAZ at age *i* and test score at age 12 y is partitioned into direct and indirect pathways. Decomposing the total effects sheds light on the pathways through which each association arises. For example, nutritional status at age 1 y may have a direct association with test scores at age 12 y, or the association may be mediated through growth at older ages. Although estimates from the path analysis are presented as direct, indirect, and total effects in keeping with the path analysis framework, we caution that these are estimates and do not necessarily represent causality. We refer to measures as HAZ at ages 1, 5, 8, and 12 y for simplicity, although the results reflect the change in HAZ from conception to age 1 y, change in HAZ from age 1 y to 5 y, etc.

We also used conditional regression, because many others have analyzed this type of data via this approach (17, 20, 29). We provide these results for consistency with earlier work and to further illustrate the relation between path analysis and conditional regression highlighted by Georgiadis et al. (16). As described elsewhere (29), we derived residuals by regressing HAZ at a given age on all previous HAZ measures [e.g., conditional HAZ (cHAZ) at 12 y is derived by regressing HAZ at 12 y on HAZ at 8 y, HAZ at 5 y, and HAZ at 1 y]. Creating conditionals removes correlations between repeated measurements and permits the inclusion of serial measures in the same regression model. Fixed effects on the child that are stable over time (e.g., maternal height) are captured by the anchor measure, HAZ at 1 y. cHAZ represents the deviation from an individual's expected HAZ based on an individual's previous HAZ measures and growth patterns in the study sample. A child with a positive cHAZ at a given age is taller than would have been predicted and a child with a negative cHAZ is shorter than would have been predicted. Thus, cHAZ can be interpreted as relative linear growth between 2 ages.

#### Sensitivity analysis

We used full information maximum likelihood (FIML) to conduct a sensitivity analysis. Children included in the sensitivity analysis were age 6–18 mo at enrollment and had ≤1 missing or outlier HAZ value as described previously. The prevalence of missing HAZ at 1, 5, 8, and 12 y was 3.1%, 0.8%, 0.9%, and 2.0%, respectively. Paternal schooling was missing for 16.4% and 20.7% in Ethiopia and Peru, respectively. In India, language of schooling instruction was missing for 25.3%. For other covariates the prevalence of missing values did not exceed 10%.

Because scores from the different tests are not directly comparable across countries, we conducted country-specific analyses. We also controlled for language of test administration within countries. In all analyses, HAZ, cHAZ, PPVT, EGRA, and Math scores were modeled as continuous variables. Models were adjusted for the control variables described, and dummy variables were created for nominal control variables with >2 categories. All analyses were conducted with the use of Stata version 14 (StataCorp LP, College Station, TX), and statistical significance was set at α = 0.05.

## Results

### 

**Tables 1** and **2** report summary statistics of language and math achievement, growth measures, and covariates. Mean HAZ in each country was <–1.00 at all ages. In all countries, >90% of children completed the PPVT in their native language or language of school instruction.

**TABLE 1:** Descriptive statistics of language and math achievement and growth measures across countries for children with HAZ at ages 1, 5, 8, and 12 y^1^

	Ethiopia		India		Peru		Vietnam	
	*n*	Mean ± SD or %	*n*	Mean ± SD or %	*n*	Mean ± SD or %	*n*	Mean ± SD or %
HAZ 1 y	1697	−1.4 ± 1.7	1777	−1.3 ± 1.4	1772	−1.3 ± 1.2	1788	−1.1 ± 1.1
HAZ 5 y	1697	−1.4 ± 1.1	1777	−1.6 ± 0.9	1772	−1.5 ± 1.1	1788	−1.3 ± 1.0
HAZ 8 y	1697	−1.2 ± 1.0	1777	−1.4 ± 1.0	1772	−1.1 ± 1.0	1788	−1.1 ± 1.0
HAZ 12 y	1697	−1.4 ± 1.0	1777	−1.4 ± 1.0	1772	−1.0 ± 1.1	1788	−1.0 ± 1.1
PPVT score	1696	33.8 ± 15.0	1777	42.9 ± 8.3	1756	86.0 ± 17.5	1788	57.9 ± 9.8
EGRA score	1416	12.9 ± 5.3	1729	13.4 ± 4.5	1754	14.5 ± 3.6	1723	14.9 ± 5.0
Math score	1482	10.5 ± 6.1	1729	12.8 ± 6.6	1754	16.2 ± 5.5	1766	16.5 ± 6.4
Language of test administration,^2^ %	1564		1327		1768		1736	
Amharic		55.2		—		—		—
Oromifa		18.9		—		—		—
Tigrigna		21.2		—		—		—
Telugu		—		48.5		—		—
English		—		48.9		—		—
Spanish		—		—		97.2		—
Vietnamese		—		—		—		99.8
Other^3^		4.7		2.6		2.8		0.2
Completed test in native language, %	1564	97.0	1777	92.2	1759	98.5	1788	91.5

^1^EGRA, Early Grades Reading Assessment; HAZ, height-for-age *z* score; PPVT, Peabody Picture Vocabulary Test.

^2^In India and Vietnam, language of instruction at child's school.

^3^In Peru, “Other” includes Quechua, Quechua and Spanish, refused to answer, and not available. In Vietnam “Other” includes Other, Chinese, and Ede.

**TABLE 2:** Descriptive characteristics across countries of children with HAZ at ages 1, 5, 8, and 12 y^1^

	Ethiopia		India		Peru		Vietnam	
	*n*	Mean ± SD or %	*n*	Mean ± SD or %	*n*	Mean ± SD or %	*n*	Mean ± SD or %
School starting age, mo	1674	91.2 ± 18.5	1758	68.3 ± 12.0	1768	74.2 ± 5.6	1776	73 ± 6.9
School type	1695		1286		1771		1775	
Public/government		87.0		58.5		81.9		97.1
Other^2^		7.9		38.0		17.8		0.8
Not enrolled		5.1		3.6		0.3		2.1
Female	1697	46.6	1777	46.4	1772	49.8	1788	48.9
Age order of siblings in the household	1676		1759		—		1786	
Eldest/middle child		0.8		0.6		—		0.3
Youngest child		74.4		43.5		—		54.1
Only child		24.8		55.9		—		45.6
Caregiver's age (y) when child was 12 y old	1693	39.1 ± 7.4	1777	34.7 ± 4.5	1769	38.0 ± 7.0	1788	38.5 ± 6.3
Caregiver's highest grade attained	1691	2.8 ± 4.5	1777	3.2 ± 4.3	1720	7.9 ± 4.5	1751	6.1 ± 4.4
Mother's height, cm	1697	158.7 ± 5.9	1777	151.6 ± 6.0	1772	150.0 ± 5.4	1788	152.2 ± 5.9
Caregiver ethnicity, %	1697		1777		1772		1788	
Amhara		29.4		—		—		—
Gurage		8.2		—		—		—
Hadia		4.7		—		—		—
Oromo		20.2		—		—		—
Sidama		5.1		—		—		—
Tigrian		21.9		—		—		—
Wolayta		6.3		—		—		—
Scheduled castes		—		17.6		—		—
Scheduled tribes		—		12.7		—		—
Backward castes		—		48.5		—		—
Open category		—		21.3		—		—
Mestizo		—		—		72.7		—
Quechua		—		—		19.0		—
White		—		—		3.4		—
Kinh		—		—		—		87.1
H'mong		—		—		—		4.5
Other^3^		4.3		—		4.9		8.5
Father's highest grade attained	1419	6.6 ± 8.4	1753	4.6 ± 5.1	1406	9.0 ± 4.0	1711	6.9 ± 4.4
Logarithm of real monthly per capita household consumption expenditure at age 12 y	1686	4.7 ± 0.6	1776	6.8 ± 0.6	1765	3.9 ± 0.8	1682	6.1 ± 0.7
Urban community at age 1 y, %	1697	35.5	1777	25.2	1772	69.2	1788	18.7

^1^Values are means ± SDs or percentages unless otherwise indicated. HAZ, height-for-age *z* score.

^2^Other includes private school, public combination (part student fees, part government-funded), nongovernmental organization/charity/religious, informal or nonformal community, charitable trust, and mix of public and private.

^3^In Peru, “Other” includes Aymara, Amazon Indian, Negro, Mulato, Zambo, and Asian/Oriental.

#### Path analysis

**Table 3** presents estimates of the direct, indirect, and total associations between HAZ at each age and language and math achievement scores at age 12 y. HAZ at each age was positively associated with each achievement test score across all 4 countries; the majority of associations were significant.

**TABLE 3:** Path analysis results for HAZ at ages 1, 5, 8, and 12 y and conditional regression results for HAZ at age 1 y and cHAZ at ages 5, 8, and 12 y, and for Math, PPVT, and EGRA scores across countries^1^

	Path analysis												
	Direct effect			Indirect effect			Total effect			Conditional regression			
	β	95% CI	*P*	β	95% CI	*P*	β	95% CI	*P*		β	95% CI	*P*
Math													
Ethiopia (*n* = 1112)													
HAZ 1 y	0.00	(−0.04, 0.04)	0.88	0.03	(0.01, 0.05)	0.01	0.03	(0.00, 0.06)	0.07	HAZ 1 y	0.03	(0.00, 0.06)	0.06
HAZ 5 y	−0.02	(−0.10, 0.05)	0.56	0.07	(0.02, 0.11)	<0.01	0.05	(−0.01, 0.11)	0.13	cHAZ 5 y	0.04	(−0.02, 0.10)	0.17
HAZ 8 y	0.09	(0.00, 0.18)	0.05	0.01	(−0.04, 0.07)	0.64	0.11	(0.04, 0.18)	<0.01	cHAZ 8 y	0.11	(0.04, 0.18)	<0.01
HAZ 12 y	0.02	(−0.07, 0.11)	0.64	—			0.02	(−0.07, 0.11)	0.64	cHAZ 12 y	0.02	(−0.07, 0.12)	0.61
India (*n* = 1279)													
HAZ 1 y	−0.01	(−0.05, 0.03)	0.66	0.06	(0.03, 0.08)	<0.01	0.05	(0.01, 0.08)	0.01	HAZ 1 y	0.05	(0.02, 0.09)	0.01
HAZ 5 y	0.04	(−0.04, 0.13)	0.35	0.09	(0.03, 0.15)	<0.01	0.13	(0.00, 0.19)	<0.01	cHAZ 5 y	0.13	(0.07, 0.19)	<0.01
HAZ 8 y	0.04	(−0.06, 0.13)	0.41	0.06	(0.01, 0.10)	0.02	0.10	(0.02, 0.18)	0.02	cHAZ 8 y	0.08	(0.00, 0.17)	0.05
HAZ 12 y	0.09	(0.02, 0.17)	0.02	—			0.09	(0.02, 0.17)	0.02	cHAZ 12 y	0.08	(0.00, 0.16)	0.05
Peru (*n* = 1370)													
HAZ 1 y	0.03	(−0.02, 0.09)	0.25	0.05	(0.02, 0.09)	0.01	0.08	(0.04, 0.13)	<0.01	HAZ 1 y	0.09	(0.05, 0.14)	<0.01
HAZ 5 y	0.09	(0.00, 0.17)	0.04	0.01	(−0.04, 0.07)	0.60	0.10	(0.04, 0.17)	<0.01	cHAZ 5 y	0.10	(0.04, 0.17)	<0.01
HAZ 8 y	−0.06	(−0.16, 0.05)	0.29	0.06	(0.00, 0.12)	0.03	0.01	(−0.08, 0.09)	0.88	cHAZ 8 y	0.01	(−0.08, 0.09)	0.88
HAZ 12 y	0.09	(0.01, 0.17)	0.03	—			0.09	(0.01, 0.17)	0.03	cHAZ 12 y	0.08	(0.00, 0.16)	0.05
Vietnam (*n* = 1569)													
HAZ 1 y	0.07	(0.01, 0.13)	0.01	0.03	(−0.01, 0.08)	0.11	0.11	(0.07, 0.15)	<0.01	HAZ 1 y	0.11	(0.07, 0.15)	<0.01
HAZ 5 y	−0.07	(−0.17, 0.04)	0.22	0.13	(0.05, 0.21)	<0.01	0.06	(−0.01, 0.13)	0.08	cHAZ 5 y	0.06	(−0.01, 0.13)	0.11
HAZ 8 y	0.04	(−0.05, 0.13)	0.39	0.06	(0.02, 0.09)	<0.01	0.10	(0.01, 0.18)	0.03	cHAZ 8 y	0.08	(0.00, 0.17)	0.06
HAZ 12 y	0.11	(0.04, 0.18)	<0.01	—			0.11	(0.04, 0.18)	<0.01	cHAZ 12 y	0.11	(0.04, 0.18)	<0.01
PPVT													
Ethiopia (*n* = 1267)													
HAZ 1 y	0.00	(−0.02, 0.02)	0.87	0.03	(0.02, 0.04)	<0.01	0.03	(0.01, 0.04)	0.01	HAZ 1 y	0.03	(0.01, 0.04)	<0.01
HAZ 5 y	0.00	(−0.04, 0.04)	1.00	0.05	(0.03, 0.08)	<0.01	0.05	(0.02, 0.09)	<0.01	cHAZ 5 y	0.05	(0.02, 0.09)	<0.01
HAZ 8 y	0.06	(0.01, 0.11)	0.02	0.02	(−0.01, 0.05)	0.16	0.08	(0.04, 0.12)	<0.01	cHAZ 8 y	0.08	(0.04, 0.11)	<0.01
HAZ 12 y	0.03	(−0.01, 0.08)	0.16	—			0.03	(−0.01, 0.08)	0.16	cHAZ 12 y	0.03	(−0.01, 0.08)	0.16
India (*n* = 1296)													
HAZ 1 y	0.03	(−0.01, 0.07)	0.18	0.04	(0.01, 0.06)	<0.01	0.06	(0.03, 0.10)	<0.01	HAZ 1 y	0.07	(0.03, 0.10)	<0.01
HAZ 5 y	0.01	(−0.07, 0.09)	0.81	0.06	(0.01, 0.12)	0.03	0.07	(0.02, 0.13)	0.01	cHAZ 5 y	0.07	(0.02, 0.13)	0.01
HAZ 8 y	0.07	(−0.02, 0.16)	0.15	0.02	(−0.03, 0.06)	0.43	0.08	(0.01, 0.16)	0.04	cHAZ 8 y	0.08	(0.00, 0.16)	0.06
HAZ 12 y	0.03	(−0.04, 0.10)	0.43	—			0.03	(−0.04, 0.10)	0.43	cHAZ 12 y	0.02	(−0.05, 0.10)	0.56
Peru (*n* = 1374)													
HAZ 1 y	0.02	(−0.04, 0.07)	0.55	0.09	(0.05, 0.12)	<0.01	0.10	(0.06, 0.14)	<0.01	HAZ 1 y	0.11	(0.07, 0.15)	<0.01
HAZ 5 y	0.07	(0.00, 0.15)	0.06	0.07	(0.02, 0.11)	0.01	0.14	(0.08, 0.20)	<0.01	cHAZ 5 y	0.14	(0.09, 0.20)	<0.01
HAZ 8 y	0.02	(−0.07, 0.12)	0.65	0.07	(0.02, 0.12)	0.01	0.09	(0.01, 0.17)	0.02	cHAZ 8 y	0.09	(0.01, 0.17)	0.02
HAZ 12 y	0.09	(0.02, 0.17)	0.01	—			0.09	(0.02, 0.17)	0.01	cHAZ 12 y	0.08	(0.01, 0.15)	0.03
Vietnam (*n* = 1589)													
HAZ 1 y	0.00	(−0.06, 0.06)	0.98	0.05	(0.01, 0.09)	0.01	0.05	(0.01, 0.09)	0.01	HAZ 1 y	0.06	(0.02, 0.10)	0.01
HAZ 5 y	−0.04	(−0.14, 0.06)	0.48	0.13	(0.05, 0.20)	<0.01	0.09	(0.01, 0.16)	0.01	cHAZ 5 y	0.09	(0.02, 0.16)	0.01
HAZ 8 y	0.07	(−0.02, 0.16)	0.13	0.04	(0.01, 0.07)	0.01	0.11	(0.01, 0.19)	0.01	cHAZ 8 y	0.10	(0.02, 0.18)	0.02
HAZ 12 y	0.08	(0.02, 0.15)	0.01	—			0.08	(0.01, 0.15)	0.01	cHAZ 12 y	0.08	(0.01, 0.14)	0.02
EGRA													
Ethiopia (*n* = 1065)													
HAZ 1 y	0.03	(−0.01, 0.07)	0.11	0.02	(0.00, 0.04)	0.08	0.05	(0.02, 0.08)	<0.01	HAZ 1 y	0.05	(0.02, 0.08)	<0.01
HAZ 5 y	−0.02	(−0.10, 0.05)	0.51	0.05	(0.01, 0.09)	0.02	0.03	(−0.03, 0.08)	0.38	cHAZ 5 y	0.03	(−0.03, 0.09)	0.37
HAZ 8 y	0.06	(−0.03, 0.15)	0.16	0.02	(−0.04, 0.07)	0.57	0.08	(0.01, 0.15)	0.03	cHAZ 8 y	0.08	(0.01, 0.15)	0.03
HAZ 12 y	0.03	(−0.06, 0.11)	0.57	—			0.03	(−0.06, 0.11)	0.57	cHAZ 12 y	0.02	(−0.06, 0.11)	0.58
India (*n* = 1290)													
HAZ 1 y	0.01	(−0.03, 0.05)	0.49	0.05	(0.03, 0.07)	<0.01	0.06	(0.03, 0.10)	<0.01	HAZ 1 y	0.07	(0.04, 0.10)	<0.01
HAZ 5 y	0.05	(−0.02, 0.13)	0.18	0.07	(0.01, 0.12)	0.01	0.12	(0.06, 0.17)	<0.01	cHAZ 5 y	0.11	(0.06, 0.17)	<0.01
HAZ 8 y	0.01	(−0.07, 0.09)	0.83	0.06	(0.01, 0.10)	0.01	0.06	(−0.01, 0.14)	0.08	cHAZ 8 y	0.05	(−0.02, 0.12)	0.18
HAZ 12 y	0.09	(0.02, 0.16)	0.01	—			0.09	(0.02, 0.16)	0.01	cHAZ 12 y	0.08	(0.01, 0.15)	0.02
Peru (*n* = 1370)													
HAZ 1 y	0.02	(−0.04, 0.08)	0.53	0.08	(0.05, 0.12)	<0.01	0.10	(0.06, 0.15)	<0.01	HAZ 1 y	0.11	(0.07, 0.16)	<0.01
HAZ 5 y	0.13	(0.05, 0.22)	0.00	0.03	(−0.02, 0.08)	0.28	0.16	(0.10, 0.23)	<0.01	cHAZ 5 y	0.16	(0.10, 0.23)	<0.01
HAZ 8 y	−0.01	(−0.11, 0.10)	0.92	0.04	(−0.02, 0.10)	0.15	0.04	(−0.05, 0.12)	0.40	cHAZ 8 y	0.04	(−0.05, 0.12)	0.40
HAZ 12 y	0.06	(−0.02, 0.14)	0.15	—			0.06	(−0.02, 0.14)	0.15	cHAZ 12 y	0.05	(−0.03, 0.13)	0.27
Vietnam (*n* = 1531)													
HAZ 1 y	0.03	(−0.03, 0.10)	0.29	0.02	(−0.03, 0.06)	0.49	0.05	(0.00, 0.10)	0.03	HAZ 1 y	0.05	(0.01, 0.10)	0.03
HAZ 5 y	−0.05	(−0.16, 0.06)	0.41	0.07	(−0.01, 0.16)	0.08	0.03	(−0.05, 0.10)	0.46	cHAZ 5 y	0.02	(−0.05, 0.10)	0.54
HAZ 8 y	0.04	(−0.06, 0.13)	0.44	0.02	(−0.01, 0.06)	0.18	0.06	(−0.03, 0.15)	0.17	cHAZ 8 y	0.06	(−0.03, 0.15)	0.21
HAZ 12 y	0.05	(−0.02, 0.12)	0.18	—			0.05	(−0.02, 0.12)	0.18	cHAZ 12 y	0.05	(−0.02, 0.12)	0.18

^1^All models adjusted for child sex, caregiver age at R4, caregiver ethnicity, caregiver years of schooling, maternal height, paternal years of schooling, natural log of R4 household expenditure per capita, and location at R1, R2, R3, and R4. In addition, Ethiopia adjusted for Young Lives child's birth order and language of test administration; India adjusted for language of school instruction; and Peru adjusted for language of test administration. cHAZ, conditional height-for-age *z* score; EGRA, Early Grades Reading Assessment; HAZ, height-for-age *z* score; PPVT, Peabody Picture Vocabulary Test; R, round.

#### HAZ at 1 y

The total effect of HAZ at 1 y on achievement test scores was positive and significant in 11 of the 12 models (3 achievement tests in 4 countries). One of the 12 direct effects was significant, whereas 9 of 12 indirect effects through HAZ at 5, 8, or 12 y were significant (see Figure 1). Among the 11 models with significant total effects for HAZ at 1 y, the proportions of the total effects mediated through HAZ at subsequent periods ranged from 31% to 60% for Math, 55% to 98% for PPVT, and 31% to 82% for EGRA. For 1 test, the positive total effect was the result of a negative direct effect and a larger positive indirect effect.

#### HAZ at 5 y

The total effect of HAZ at 5 y on achievement test scores was positive and significant in 8 of 12 models. Two of the 12 direct effects were significant, whereas 9 of 12 indirect effects through HAZ at 8 and 12 y were significant. Among the 8 models with significant total effects for HAZ at age 5 y, the proportions of the total effects mediated through HAZ at subsequent periods ranged from 14% to 68% for Math, 47% to 100% for PPVT, and 18% to 56% for EGRA. For 5 tests, the positive total effect was the result of a negative direct effect and a larger positive indirect effect.

#### HAZ at 8 y

The total effect of HAZ at age 8 y on achievement test scores was positive and significant in 8 of the 12 models. Two of the 12 direct effects were significant, whereas 6 of 12 indirect effects through HAZ at age 12 y were significant. Among the 8 models with significant total effects for HAZ at age 8 y, the proportions of the total effects mediated through HAZ at subsequent periods ranged from 13% to 59% for Math, 22% to 75% for PPVT, and 20% for EGRA. For 2 tests, the positive total effect was the result of a negative direct effect and a larger positive indirect effect.

#### HAZ at 12 y

The total effect of HAZ at age 12 y on achievement test scores was positive and significant in 6 of the 12 models. These significant direct effects were observed in 3 Math models, 2 PPVT models, and 1 EGRA model.

#### Model consistency with variation in test scores

The model consistency with variation in test scores ranged from *r^2^* = 3.5% to *r^2^* = 14.8% for Math, *r^2^* = 8.5% to *r^2^* = 16.0% for PPVT, and *r^2^* = 3.8% to *r^2^* = 18.3% for EGRA (**Table 4**). Most of this consistency was attributable to covariates; as a set, the 4 HAZ measures were consistent with *r^2^* = 0.4–3.5% of the variances. HAZ at ages 1 and 5 y were more consistent with the variation in test scores than HAZ at ages 8 and 12 y, with the exceptions of Math in Ethiopia and PPVT and EGRA in Vietnam.

**TABLE 4:** Model consistency with variation in Math, PPVT, and EGRA scores attributable to the full path models, HAZ at ages 1, 5, 8, and 12 y, and all HAZ measures combined^1^

	Full model, %	HAZ 1 y, %	HAZ 5 y, %	HAZ 8 y, %	HAZ 12 y, %	HAZ at all ages, %
Math						
Ethiopia	3.5	0.3	0.2	0.9	−0.2	1.3
India	14.8	0.6	1.1	0.4	0.4	2.4
Peru	10.2	1.1	0.3	−0.1	0.5	1.7
Vietnam	8.9	1.3	0.2	0.2	0.8	2.6
PPVT						
Ethiopia	8.5	0.8	0.8	1.2	0.1	2.9
India	11.4	1.6	0.4	0.3	0.1	2.4
Peru	16.0	1.5	1.2	0.3	0.5	3.5
Vietnam	10.8	−0.2	0.6	0.2	0.4	1.0
EGRA						
Ethiopia	3.8	0.8	0.1	0.6	0.0	1.5
India	18.3	1.3	1.3	0.3	0.6	3.4
Peru	11.8	1.2	1.6	−0.1	0.1	2.8
Vietnam	7.2	0.1	0.0	0.0	0.3	0.4

^1^All models adjusted for child sex, caregiver age at R4, caregiver ethnicity, caregiver years of schooling, maternal height, paternal years of schooling, natural log of R4 household expenditure per capita, and location at R1, R2, R3, and R4. In addition, Ethiopia adjusted for Young Lives child's birth order and language of test administration; India adjusted for language of school instruction; and Peru adjusted for language of test administration. EGRA, Early Grades Reading Assessment; HAZ, height-for-age *z* score; PPVT, Peabody Picture Vocabulary Test; R, round.

#### Conditional regressions

Results from conditional regressions were equivalent to the total effects of the path models with some rounding error (Table 3). HAZ at age 1 y was positively associated with all 3 test scores across all 4 countries and significantly associated in 11 of the 12 models. The coefficients for cHAZ at age 5 y were positive for all 12 associations examined and significant for 8. The coefficients for cHAZ at age 8 y were positive for all age 12 associations and significant for 5. The coefficients for cHAZ at age 12 y were positive for all 12 associations and significant for 4.

#### Sensitivity analysis

Comparing participants included in the main results with those added in the FIML results, the additional participants had lower HAZ, test scores, caregiver schooling attainment, and household expenditures, and were more likely to belong to minority ethnic groups (**Supplemental Tables 1 and 2**). However, findings from the FIML analysis were consistent with the findings from the main results, although there were minor differences in the estimates (**Supplemental Table 3**). Two total effects that were significant in the main results were no longer significant in the sensitivity analysis: the association of HAZ at age 8 y with PPVT score in India and the association of HAZ at age 8 y with EGRA score in Ethiopia. Two total effects were significant in the sensitivity analysis but not in the main analysis: the associations of HAZ at ages 1 and 5 y with Math score in Ethiopia. Two direct effects were significant in the FIML results but not the main analysis: the association of HAZ at age 8 y with Math score in Ethiopia and of HAZ at 1 y and the PPVT score in India. Finally, 1 indirect effect was significant in the main results but not in the sensitivity analysis: the association of HAZ at age 5 y with EGRA score in Ethiopia.

In the conditional regression models, 3 associations that were significant in the main results were not significant in the FIML results: the association of cHAZ at age 5 y with PPVT score in India, the association of cHAZ at age 8 y with EGRA score in Ethiopia, and the association of HAZ at age 1 y with EGRA score in Vietnam. Three associations that were not significant in the main results were significant in the FIML results: the associations of HAZ at age 1 y and cHAZ at age 5 y with Math score in Ethiopia and the association of cHAZ at age 12 y with Math score in Peru.

## Discussion

We used data from the YL Younger Cohort to examine whether relative linear growth from early life through early adolescence was associated with language and math achievement in early adolescence. We examined the association of HAZ with 3 achievement test scores across 4 countries, for a total of 12 models at each age. HAZ at age 1 y was positively and significantly associated with achievement scores in 11 of the 12 models examined. HAZ at ages 5, 8, and 12 y were positively and significantly associated with achievement scores in 8, 8, and 6 of the 12 models, respectively. The associations of HAZ at ages 1 and 5 y with achievement scores were each mediated through HAZ at subsequent periods in 9 of the 12 models. The associations of HAZ at age 8 y with achievement scores were mediated through HAZ at age 12 y in 6 of the 12 models.

Our analysis extends prior examinations and demonstrates that at age 12 y, children who grew faster than their peers between any 2 ages performed better on language and math assessments. This was consistent across tests and countries. A larger number of these associations were statistically significant at younger ages (11 of 12 associations for HAZ at age 1 y and 8 of 12 for HAZ at age 5 y) than at older ages (6 of 12 for HAZ at age 12 y). Consistent with the earlier findings at age 8 y, the associations of HAZ at ages 1 and 5 y with language and math achievement scores were largely mediated through HAZ at older ages (16). In our analysis, the associations of HAZ at age 8 y with achievement scores were mediated through HAZ at 12 y in half of the models examined.

Statistical significance aside, only small proportions of the variations in language and math achievement scores were attributable to linear growth in these 4 different country contexts. This suggests that after early life, where the literature suggests the associations between height and language and math achievement are strong, attributes of the community, such as schooling, play an increasing role in language and math achievement relative to growth. In early life, nutrition may be a direct cause of cognitive development, because nutrition and stimulation are intertwined through breastfeeding and complementary feeding. Later in childhood, feeding and stimulation are increasingly decoupled as opportunities for stimulation increasingly occur outside the home.

We also further illustrate the relation between path analysis and conditional regression highlighted by Georgiadis et al. (16), and show that path analysis and conditional regression yield consistent results. These approaches are mathematically related and reflect different approaches to partitioning the same variance in HAZ at a given age and its association with language and math achievement scores (13). Conditionals are a special case of path models in which a structural constraint is placed on the indirect paths by setting them to 0, therefore the β coefficient for a conditional is identical, give or take rounding error, to the “total effect” in a path model. An advantage of path analysis is that the variances between HAZ *i* and test scores can be decomposed into direct and indirect effects, shedding light on the pathways through which the associations arise, whether directly or mediated through HAZ at older ages. It is worth noting that, although we use HAZ measures at 4 ages, both path and conditional regression analysis approaches model the change to HAZ between 2 ages given earlier growth. Because HAZ is a standardized measure, change in HAZ therefore is a measure of relative linear growth, rather than absolute linear growth, although the inferences are the same.

Strengths of this study include the use of data from a large prospective study in 4 LMICs. The study has extensive follow-up time with low attrition and has collected a wide range of data on child growth and language and math achievement. However, the context is substantially different in the 4 countries, which affected the cross-country interpretation of the achievement measures and precluded a pooled 4-country analysis. The tests were administered in multiple languages, but we controlled for language of administration in our models. Nevertheless, the magnitudes of coefficients, patterns of significant associations, and proportions of variance explained by the models are similar across countries. The YL data are observational and 1 particular path model was specified, therefore the results do not necessarily reflect causality and should be interpreted with caution. Overall, child linear growth was a weak but consistent predictor of language and math achievement at age 12 y in 4 different LMIC contexts.

## Supplementary Material

Supplemental Figures and Tables

## Notes

Supported by Grand Challenges Canada grant 0072-03 (to JRB) and Bill and Melinda Gates Foundation grant OPP1032713 (to JRB). AJK was supported by NIH award T32 DK007734. The data used in this study come from Young Lives, an international study of childhood poverty, following the lives of 12,000 children in 4 countries—Ethiopia, India, Peru, and Vietnam—over 15 y (www.younglives.org.uk). Young Lives is core-funded by UK aid from the Department for International Development (DFID) and was co-funded from 2010 to 2014 by the Netherlands Ministry of Foreign Affairs and from 2014 to 2015 by Irish Aid.

Author disclosures: AJK, AG, JRB, BTC, LCHF, and ADS, no conflicts of interest.

Supplemental Tables 1–3 and Supplemental Figure 1 are available from the “Supplementary data” link in the online posting of the article and from the same link in the online table of contents at https://academic.oup.com/jn/.

Abbreviations used: cHAZ, conditional height-for-age *z* score; EGRA, Early Grade Reading Assessment; FIML, full information maximum likelihood; HAZ, height-for-age *z* score; LMIC, low- or middle-income country; PPVT, Peabody Picture Vocabulary Test; YL, Young Lives.
