# Supplementary material for: Linear Growth through 12 Years is Weakly but Consistently Associated with Language and Math Achievement Scores at Age 12 Years in 4 Low- or Middle-Income Countries
Source: J Nutr. 2018 Nov 1;148(11):1852–9. doi: 10.1093/jn/nxy191 (PMC6209809; doi:10.1093/jn/nxy191)
Supplement: Supplemental Figures and Tables [file nxy191_supplemental_figures_tables.docx]

**Supplemental Tables and Figures**

Supplemental Table 1. Comparison of selected characteristics for observations with complete HAZ information at ages 1, 5, 8, and 12 y and observations with incomplete HAZ information in Ethiopia and India.

Supplemental Table 2. Comparison of selected characteristics for observations with complete HAZ information at ages 1, 5, 8, and 12 y and observations with incomplete HAZ information in Peru and Vietnam.

Supplemental Table 3. Full information maximum likelihood path analysis results for HAZ at ages 1, 5, 8 and 12 y and full information maximum likelihood conditional regression results for HAZ at age 1 y and cHAZ at ages 5, 8, and 12 y and for Mathematics, PPVT, and EGRA scores across countries.

Supplemental Figure 1. Exclusions of children for missing or outlying HAZ, missing achievement test scores, or missing covariate information by country. HAZ, height-for-age z-score.

Supplemental Table 1. Comparison of selected characteristics for observations with complete HAZ information at ages 1, 5, 8, and 12 y and observations with incomplete HAZ information in Ethiopia and India.^1^

|  | Ethiopia | | | | |  | India | | | | |
| --- | --- | --- | --- | --- | --- | --- | --- | --- | --- | --- | --- |
|  | Complete HAZ | | Incomplete HAZ | |  |  | Complete HAZ | | Incomplete HAZ | |  |
|  | *n* |  | *n* |  | *p* |  | *n* |  | *n* |  | *p* |
| HAZ 1 y | 1697 | -1.4 ± 1.7 | 124 | -2.8 ± 3.8 | <0.01 |  | 1777 | -1.3 ± 1.4 | 96 | -1.6 ± 3.7 | 0.10 |
| HAZ 5 y | 1697 | -1.4 ± 1.1 | 163 | -1.5 ± 1.5 | 0.42 |  | 1777 | -1.6 ± 0.9 | 99 | -1.9 ± 1.2 | 0.02 |
| HAZ 8 y | 1697 | -1.2 ± 1.0 | 156 | -1.5 ± 1.4 | 0.03 |  | 1777 | -1.4 ± 1.0 | 101 | -1.8 ± 1.3 | 0.01 |
| HAZ 12 y | 1697 | -1.4 ± 1.0 | 154 | -1.6 ± 1.2 | 0.19 |  | 1777 | -1.4 ± 1.0 | 85 | -1.8 ± 1.2 | <0.01 |
| PPVT score | 1696 | 33.8 ± 15.0 | 157 | 32.7 ± 16.1 | 0.37 |  | 1777 | 42.9 ± 8.3 | 94 | 41.6 ± 9.9 | 0.20 |
| Reading score | 1416 | 12.9 ± 5.3 | 135 | 12.2 ± 5.0 | 0.13 |  | 1729 | 13.4 ± 4.5 | 88 | 13.1 ± 4.3 | 0.59 |
| Math score | 1482 | 10.5 ± 6.1 | 141 | 9.9 ± 5.6 | 0.27 |  | 1729 | 12.8 ± 6.6 | 88 | 11.9 ± 6.8 | 0.20 |
| Caregiver's highest grade attained | 1691 | 2.8 ± 4.5 | 168 | 2.9 ± 4.9 | 0.72 |  | 1777 | 3.2 ± 4.3 | 112 | 2.2 ± 3.7 | 0.01 |
| Caregiver ethnicity, % | 1697 |  | 168 |  | 0.52 |  | 1777 |  | 113 |  | 0.60 |
| Amhara |  | 29.4 |  | 30.4 |  |  |  |  |  |  |  |
| Gurage |  | 8.2 |  | 5.4 |  |  |  |  |  |  |  |
| Hadia |  | 4.7 |  | 4.8 |  |  |  |  |  |  |  |
| Oromo |  | 20.2 |  | 19.1 |  |  |  |  |  |  |  |
| Sidama |  | 5.1 |  | 5.1 |  |  |  |  |  |  |  |
| Tigrian |  | 21.9 |  | 21.9 |  |  |  |  |  |  |  |
| Wolayta |  | 6.3 |  | 6.3 |  |  |  |  |  |  |  |
| Scheduled castes |  |  |  |  |  |  |  | 17.6 |  | 18.6 |  |
| Scheduled tribes |  |  |  |  |  |  |  | 12.7 |  | 16.8 |  |
| Backward castes |  |  |  |  |  |  |  | 48.5 |  | 44.3 |  |
| Open category |  |  |  |  |  |  |  | 21.3 |  | 20.4 |  |
| Other^2^ |  | 4.3 |  | 3.6 |  |  |  | . |  |  |  |
| Logarithm of real monthly per capita household consumption expenditure at age 12 y | 1686 | 4.7 ± 0.6 | 155 | 4.7 ± 0.7 | 0.61 |  | 1776 | 6.8 ± 0.6 | 94 | 6.7 ± 0.6 | 0.03 |
| ^1^Values are means ± SDs unless otherwise indicated. EGRA, Early Grades Reading Assessment; HAZ, height-for-age z-score; PPVT, Peabody Picture Vocabulary Test.  ^2^In Peru 'Other' includes Aymara, Amazon Indian, Negro, Mulato, Zambo, and Asian/Oriental. | | | | | | | | | | | |

Supplemental Table 2. Comparison of selected characteristics for observations with complete HAZ information at ages 1, 5, 8, and 12 y and observations with incomplete HAZ information in Peru and Vietnam.^1^

|  | Peru | | | | |  | Vietnam | | | | |
| --- | --- | --- | --- | --- | --- | --- | --- | --- | --- | --- | --- |
|  | Complete HAZ | | Incomplete HAZ | |  |  | Complete HAZ | | Incomplete HAZ | |  |
|  | *n* |  | *n* |  | *p* |  | *n* |  | *n* |  | *p* |
| HAZ 1 y | 1772 | -1.3 ± 1.2 | 129 | -1.5 ± 2.4 | 0.36 |  | 1788 | -1.1 ± 1.1 | 89 | -0.9 ± 2.1 | 0.12 |
| HAZ 5 y | 1772 | -1.5 ± 1.1 | 109 | -1.9 ± 1.2 | <0.01 |  | 1788 | -1.3 ± 1.0 | 81 | -1.2 ± 1.3 | 0.27 |
| HAZ 8 y | 1772 | -1.1 ± 1.0 | 124 | -1.5 ± 1.2 | <0.01 |  | 1788 | -1.1 ± 1.0 | 66 | -1.0 ± 1.3 | 0.42 |
| HAZ 12 y | 1772 | -1.0 ± 1.1 | 73 | -1.6 ± 1.2 | <0.01 |  | 1788 | -1.0 ± 1.1 | 50 | -0.8 ± 1.4 | 0.50 |
| PPVT score | 1756 | 86.0 ± 17.5 | 90 | 81.3 ± 17.1 | 0.01 |  | 1788 | 57.9 ± 9.8 | 59 | 52.3 ± 17.6 | 0.02 |
| Reading score | 1754 | 14.5 ± 3.6 | 88 | 13.4 ± 3.3 | 0.01 |  | 1723 | 14.9 ± 5.0 | 57 | 13.9 ± 5.8 | 0.14 |
| Math score | 1754 | 16.2 ± 5.5 | 88 | 15.2 ± 5.6 | 0.09 |  | 1766 | 16.5 ± 6.4 | 59 | 14.9 ± 7.3 | 0.06 |
| Caregiver's highest grade attained | 1720 | 7.9 ± 4.5 | 132 | 6.8 ± 4.7 | <0.01 |  | 1751 | 6.1 ± 4.4 | 89 | 7.0 ± 4.6 | 0.07 |
| Caregiver ethnicity, % | 1772 |  | 139 |  | 0.01 |  | 1788 |  | 92 |  | 0.01 |
| Mestizo |  | 72.7 |  | 61.9 |  |  |  |  |  |  |  |
| Quechua |  | 19.0 |  | 29.5 |  |  |  |  |  |  |  |
| White |  | 3.4 |  | 5.0 |  |  |  |  |  |  |  |
| Kinh |  |  |  |  |  |  |  | 87.1 |  | 83.7 |  |
| H'mong |  |  |  |  |  |  |  | 4.5 |  | 10.9 |  |
| Other^2^ |  | 4.9 |  | 3.6 |  |  |  | 8.5 |  | 5.4 |  |
| Logarithm of real monthly per capita household consumption expenditure at age 12 y | 1765 | 3.9 ± 0.8 | 84 | 3.6 ± 0.8 | 0.01 |  | 1682 | 6.1 ± 0.7 | 54 | 6.1 ± 0.8 | 0.98 |
| ^1^For each country, values in the first column are *n* for the characteristic and values in the second column are means ± SDs or percentages for the observations with complete HAZ information. These values for observations with incomplete HAZ information are in the third and fourth columns for each country, respectively. EGRA, Early Grades Reading Assessment; HAZ, height-for-age z-score; PPVT, Peabody Picture Vocabulary Test.  ^2^In Peru 'Other' includes Aymara, Amazon Indian, Negro, Mulato, Zambo, and Asian/Oriental. | | | | | | | | | | | |

Supplemental Table 3. Full information maximum likelihood path analysis results for HAZ at ages 1, 5, 8 and 12 y and full information maximum likelihood conditional regression results for HAZ at age 1 y and cHAZ at ages 5, 8, and 12 y and for Mathematics, PPVT, and EGRA scores across countries.

|  |  | Path analysis | |  |  |  |  |  |  |  |  |  |  | Conditional regression | |  |  |
| --- | --- | --- | --- | --- | --- | --- | --- | --- | --- | --- | --- | --- | --- | --- | --- | --- | --- |
|  |  | Direct effect | |  |  | Indirect effect | |  |  | Total effect | |  |  |  |  |  |  |
|  |  | β | 95% CI | p-value |  | β | 95% CI | p-value |  | β | 95% CI | p-value |  |  | β | 95% CI | p-value |
| Math |  |  |  |  |  |  |  |  |  |  |  |  |  |  |  |  |  |
| Ethiopia (*n*=1865) |  |  |  |  |  |  |  |  |  |  |  |  |  |  |  |  |  |
| HAZ 1 y |  | 0.01 | (-0.03, 0.04) | 0.69 |  | 0.03 | (0.01, 0.05) | <0.01 |  | 0.04 | (0.01, 0.07) | <0.01 |  | HAZ 1 y | 0.04 | (0.01, 0.07) | <0.01 |
| HAZ 5 y |  | 0.00 | (-0.06, 0.06) | 0.94 |  | 0.06 | (0.03, 0.10) | <0.01 |  | 0.06 | (0.01, 0.11) | 0.01 |  | cHAZ 5 y | 0.06 | (0.01, 0.11) | 0.03 |
| HAZ 8 y |  | 0.10 | (0.02, 0.17) | 0.01 |  | 0.01 | (-0.04, 0.05) | 0.78 |  | 0.10 | (0.04, 0.16) | <0.01 |  | cHAZ 8 y | 0.12 | (0.06, 0.19) | <0.01 |
| HAZ 12 y |  | 0.01 | (-0.06, 0.09) | 0.78 |  | -- |  |  |  | 0.01 | (-0.06, 0.09) | 0.78 |  | cHAZ 12 y | 0.00 | (-0.07, 0.08) | 0.92 |
|  |  |  |  |  |  |  |  |  |  |  |  |  |  |  |  |  |  |
| India (*n*=1890) |  |  |  |  |  |  |  |  |  |  |  |  |  |  |  |  |  |
| HAZ 1 y |  | 0.01 | (-0.03, 0.05) | 0.65 |  | 0.05 | (0.03, 0.08) | <0.01 |  | 0.06 | (0.03, 0.09) | <0.01 |  | HAZ 1 y | 0.07 | (0.03, 0.10) | <0.01 |
| HAZ 5 y |  | 0.05 | (-0.03, 0.12) | 0.23 |  | 0.07 | (0.02, 0.13) | 0.01 |  | 0.12 | (0.07, 0.17) | <0.01 |  | cHAZ 5 y | 0.12 | (0.06, 0.17) | <0.01 |
| HAZ 8 y |  | 0.04 | (-0.04, 0.12) | 0.35 |  | 0.04 | (0.00, 0.08) | 0.04 |  | 0.08 | (0.01, 0.15) | 0.03 |  | cHAZ 8 y | 0.07 | (0.00, 0.14) | 0.06 |
| HAZ 12 y |  | 0.07 | (0.00, 0.14) | 0.04 |  | -- |  |  |  | 0.07 | (0.00, 0.14) | 0.04 |  | cHAZ 12 y | 0.07 | (0.00, 0.14) | 0.05 |
|  |  |  |  |  |  |  |  |  |  |  |  |  |  |  |  |  |  |
| Peru (*n*=1911) |  |  |  |  |  |  |  |  |  |  |  |  |  |  |  |  |  |
| HAZ 1 y |  | 0.03 | (-0.02, 0.08) | 0.31 |  | 0.06 | (0.03, 0.09) | <0.01 |  | 0.09 | (0.05, 0.13) | <0.01 |  | HAZ 1 y | 0.09 | (0.05, 0.13) | <0.01 |
| HAZ 5 y |  | 0.09 | (0.02, 0.17) | 0.01 |  | 0.03 | (-0.02, 0.07) | 0.25 |  | 0.12 | (0.06, 0.18) | <0.01 |  | cHAZ 5 y | 0.12 | (0.06, 0.17) | <0.01 |
| HAZ 8 y |  | -0.03 | (-0.11, 0.06) | 0.58 |  | 0.05 | (0.00, 0.10) | 0.04 |  | 0.03 | (-0.05, 0.10) | 0.47 |  | cHAZ 8 y | 0.03 | (-0.04, 0.11) | 0.42 |
| HAZ 12 y |  | 0.07 | (0.00, 0.14) | 0.04 |  | -- |  |  |  | 0.07 | (0.00, 0.14) | 0.04 |  | cHAZ 12 y | 0.08 | (0.00, 0.15) | 0.04 |
|  |  |  |  |  |  |  |  |  |  |  |  |  |  |  |  |  |  |
| Vietnam (*n*=1880) |  |  |  |  |  |  |  |  |  |  |  |  |  |  |  |  |  |
| HAZ 1 y |  | 0.06 | (0.00, 0.11) | 0.04 |  | 0.03 | (0.00, 0.07) | 0.09 |  | 0.09 | (0.05, 0.13) | <0.01 |  | HAZ 1 y | 0.10 | (0.06, 0.14) | <0.01 |
| HAZ 5 y |  | -0.06 | (-0.16, 0.04) | 0.23 |  | 0.12 | (0.05, 0.19) | <0.01 |  | 0.06 | (0.00, 0.13) | 0.06 |  | cHAZ 5 y | 0.06 | (0.00, 0.13) | 0.07 |
| HAZ 8 y |  | 0.03 | (-0.05, 0.11) | 0.48 |  | 0.05 | (0.02, 0.08) | <0.01 |  | 0.08 | (0.00, 0.15) | 0.04 |  | cHAZ 8 y | 0.07 | (-0.01, 0.15) | 0.08 |
| HAZ 12 y |  | 0.11 | (0.05, 0.18) | <0.01 |  | -- |  |  |  | 0.11 | (0.05, 0.18) | <0.01 |  | cHAZ 12 y | 0.11 | (0.05, 0.18) | <0.01 |
|  |  |  |  |  |  |  |  |  |  |  |  |  |  |  |  |  |  |
| PPVT |  |  |  |  |  |  |  |  |  |  |  |  |  |  |  |  |  |
| Ethiopia (*n*=1865) |  |  |  |  |  |  |  |  |  |  |  |  |  |  |  |  |  |
| HAZ 1 y |  | 0.00 | (-0.02, 0.02) | 0.73 |  | 0.03 | (0.02, 0.04) | <0.01 |  | 0.02 | (0.01, 0.04) | <0.01 |  | HAZ 1 y | 0.03 | (0.01, 0.04) | <0.01 |
| HAZ 5 y |  | 0.01 | (-0.02, 0.05) | 0.48 |  | 0.04 | (0.02, 0.06) | <0.01 |  | 0.06 | (0.03, 0.08) | <0.01 |  | cHAZ 5 y | 0.05 | (0.03, 0.08) | <0.01 |
| HAZ 8 y |  | 0.06 | (0.02, 0.10) | <0.01 |  | 0.01 | (-0.02, 0.03) | 0.59 |  | 0.07 | (0.04, 0.10) | <0.01 |  | cHAZ 8 y | 0.08 | (0.04, 0.11) | <0.01 |
| HAZ 12 y |  | 0.01 | (-0.03, 0.05) | 0.59 |  | -- |  |  |  | 0.01 | (-0.03, 0.05) | 0.59 |  | cHAZ 12 y | 0.02 | (-0.02, 0.07) | 0.33 |
|  |  |  |  |  |  |  |  |  |  |  |  |  |  |  |  |  |  |
| India (*n*=1890) |  |  |  |  |  |  |  |  |  |  |  |  |  |  |  |  |  |
| HAZ 1 y |  | 0.06 | (0.02, 0.10) | <0.01 |  | 0.03 | (0.01, 0.05) | 0.01 |  | 0.09 | (0.06, 0.12) | <0.01 |  | HAZ 1 y | 0.09 | (0.06, 0.12) | <0.01 |
| HAZ 5 y |  | 0.01 | (-0.06, 0.09) | 0.77 |  | 0.05 | (0.00, 0.11) | 0.04 |  | 0.07 | (0.01, 0.12) | 0.02 |  | cHAZ 5 y | 0.05 | (0.00, 0.11) | 0.06 |
| HAZ 8 y |  | 0.03 | (-0.05, 0.11) | 0.44 |  | 0.03 | (-0.01, 0.07) | 0.16 |  | 0.06 | (-0.01, 0.13) | 0.10 |  | cHAZ 8 y | 0.04 | (-0.03, 0.12) | 0.23 |
| HAZ 12 y |  | 0.05 | (-0.02, 0.11) | 0.16 |  | -- |  |  |  | 0.05 | (-0.02, 0.11) | 0.16 |  | cHAZ 12 y | 0.04 | (-0.03, 0.11) | 0.23 |
|  |  |  |  |  |  |  |  |  |  |  |  |  |  |  |  |  |  |
| Peru (*n*=1911) |  |  |  |  |  |  |  |  |  |  |  |  |  |  |  |  |  |
| HAZ 1 y |  | 0.03 | (-0.01, 0.07) | 0.19 |  | 0.08 | (0.06, 0.11) | <0.01 |  | 0.11 | (0.08, 0.15) | <0.01 |  | HAZ 1 y | 0.12 | (0.09, 0.16) | <0.01 |
| HAZ 5 y |  | 0.06 | (-0.01, 0.12) | 0.07 |  | 0.07 | (0.03, 0.11) | <0.01 |  | 0.13 | (0.08, 0.18) | <0.01 |  | cHAZ 5 y | 0.13 | (0.08, 0.18) | <0.01 |
| HAZ 8 y |  | 0.03 | (-0.05, 0.11) | 0.49 |  | 0.07 | (0.03, 0.12) | <0.01 |  | 0.10 | (0.03, 0.17) | <0.01 |  | cHAZ 8 y | 0.09 | (0.03, 0.16) | 0.01 |
| HAZ 12 y |  | 0.10 | (0.04, 0.16) | <0.01 |  | -- |  |  |  | 0.10 | (0.04, 0.16) | <0.01 |  | cHAZ 12 y | 0.08 | (0.02, 0.15) | 0.01 |
|  |  |  |  |  |  |  |  |  |  |  |  |  |  |  |  |  |  |
| Vietnam (*n*=1880) |  |  |  |  |  |  |  |  |  |  |  |  |  |  |  |  |  |
| HAZ 1 y |  | 0.00 | (-0.06, 0.05) | 0.92 |  | 0.06 | (0.02, 0.10) | <0.01 |  | 0.06 | (0.02, 0.10) | <0.01 |  | HAZ 1 y | 0.07 | (0.03, 0.10) | <0.01 |
| HAZ 5 y |  | 0.00 | (-0.10, 0.09) | 0.92 |  | 0.11 | (0.04, 0.18) | <0.01 |  | 0.11 | (0.04, 0.17) | <0.01 |  | cHAZ 5 y | 0.11 | (0.04, 0.17) | <0.01 |
| HAZ 8 y |  | 0.04 | (-0.04, 0.12) | 0.35 |  | 0.04 | (0.01, 0.07) | <0.01 |  | 0.08 | (0.00, 0.15) | 0.04 |  | cHAZ 8 y | 0.08 | (0.01, 0.16) | 0.04 |
| HAZ 12 y |  | 0.09 | (0.03, 0.15) | <0.01 |  | -- |  |  |  | 0.09 | (0.03, 0.15) | <0.01 |  | cHAZ 12 y | 0.08 | (0.02, 0.14) | 0.01 |
|  |  |  |  |  |  |  |  |  |  |  |  |  |  |  |  |  |  |
| EGRA |  |  |  |  |  |  |  |  |  |  |  |  |  |  |  |  |  |
| Ethiopia (*n*=1865) |  |  |  |  |  |  |  |  |  |  |  |  |  |  |  |  |  |
| HAZ 1 y |  | 0.03 | (0.00, 0.06) | 0.05 |  | 0.02 | (0.00, 0.03) | 0.09 |  | 0.05 | (0.02, 0.07) | <0.01 |  | HAZ 1 y | 0.05 | (0.02, 0.08) | <0.01 |
| HAZ 5 y |  | -0.01 | (-0.07, 0.05) | 0.76 |  | 0.04 | (0.00, 0.07) | 0.05 |  | 0.03 | (-0.02, 0.07) | 0.27 |  | cHAZ 5 y | 0.03 | (-0.02, 0.08) | 0.28 |
| HAZ 8 y |  | 0.02 | (-0.06, 0.09) | 0.66 |  | 0.03 | (-0.01, 0.07) | 0.16 |  | 0.05 | (-0.01, 0.10) | 0.10 |  | cHAZ 8 y | 0.06 | (0.00, 0.12) | 0.07 |
| HAZ 12 y |  | 0.05 | (-0.02, 0.12) | 0.16 |  | -- |  |  |  | 0.05 | (-0.02, 0.12) | 0.16 |  | cHAZ 12 y | 0.04 | (-0.03, 0.12) | 0.24 |
|  |  |  |  |  |  |  |  |  |  |  |  |  |  |  |  |  |  |
| India (*n*=1890) |  |  |  |  |  |  |  |  |  |  |  |  |  |  |  |  |  |
| HAZ 1 y |  | 0.03 | (-0.01, 0.06) | 0.14 |  | 0.05 | (0.03, 0.07) | <0.01 |  | 0.07 | (0.04, 0.10) | <0.01 |  | HAZ 1 y | 0.07 | (0.05, 0.10) | <0.01 |
| HAZ 5 y |  | 0.06 | (-0.01, 0.12) | 0.10 |  | 0.05 | (0.00, 0.10) | 0.04 |  | 0.11 | (0.06, 0.16) | <0.01 |  | cHAZ 5 y | 0.10 | (0.05, 0.15) | <0.01 |
| HAZ 8 y |  | 0.00 | (-0.07, 0.07) | 1.00 |  | 0.04 | (0.01, 0.08) | 0.02 |  | 0.04 | (-0.02, 0.11) | 0.18 |  | cHAZ 8 y | 0.03 | (-0.04, 0.09) | 0.43 |
| HAZ 12 y |  | 0.08 | (0.02, 0.14) | 0.01 |  | -- |  |  |  | 0.08 | (0.02, 0.14) | 0.01 |  | cHAZ 12 y | 0.08 | (0.02, 0.14) | 0.01 |
|  |  |  |  |  |  |  |  |  |  |  |  |  |  |  |  |  |  |
| Peru (*n*=1911) |  |  |  |  |  |  |  |  |  |  |  |  |  |  |  |  |  |
| HAZ 1 y |  | 0.02 | (-0.03, 0.07) | 0.35 |  | 0.08 | (0.05, 0.12) | <0.01 |  | 0.11 | (0.07, 0.15) | <0.01 |  | HAZ 1 y | 0.12 | (0.08, 0.16) | <0.01 |
| HAZ 5 y |  | 0.12 | (0.05, 0.20) | <0.01 |  | 0.03 | (-0.01, 0.08) | 0.14 |  | 0.16 | (0.10, 0.21) | <0.01 |  | cHAZ 5 y | 0.16 | (0.10, 0.21) | <0.01 |
| HAZ 8 y |  | 0.01 | (-0.08, 0.10) | 0.77 |  | 0.03 | (-0.02, 0.08) | 0.19 |  | 0.05 | (-0.03, 0.12) | 0.22 |  | cHAZ 8 y | 0.04 | (-0.03, 0.12) | 0.27 |
| HAZ 12 y |  | 0.05 | (-0.02, 0.11) | 0.19 |  | -- |  |  |  | 0.05 | (-0.02, 0.11) | 0.19 |  | cHAZ 12 y | 0.04 | (-0.03, 0.11) | 0.27 |
|  |  |  |  |  |  |  |  |  |  |  |  |  |  |  |  |  |  |
| Vietnam (*n*=1880) |  |  |  |  |  |  |  |  |  |  |  |  |  |  |  |  |  |
| HAZ 1 y |  | 0.02 | (-0.03, 0.08) | 0.42 |  | 0.01 | (-0.03, 0.05) | 0.58 |  | 0.04 | (-0.01, 0.08) | 0.09 |  | HAZ 1 y | 0.04 | (0.00, 0.08) | 0.08 |
| HAZ 5 y |  | -0.04 | (-0.15, 0.06) | 0.39 |  | 0.07 | (-0.01, 0.14) | 0.08 |  | 0.02 | (-0.05, 0.09) | 0.54 |  | cHAZ 5 y | 0.02 | (-0.05, 0.09) | 0.59 |
| HAZ 8 y |  | 0.03 | (-0.05, 0.11) | 0.48 |  | 0.02 | (-0.01, 0.05) | 0.16 |  | 0.05 | (-0.03, 0.13) | 0.21 |  | cHAZ 8 y | 0.04 | (-0.04, 0.12) | 0.29 |
| HAZ 12 y |  | 0.05 | (-0.02, 0.11) | 0.16 |  | -- |  |  |  | 0.05 | (-0.02, 0.11) | 0.16 |  | cHAZ 12 y | 0.05 | (-0.02, 0.12) | 0.16 |
| ^1^All models adjusted for child sex, caregiver age at R4, caregiver ethnicity, caregiver years of schooling, maternal height, paternal years of schooling, natural log of R4 household expenditure per capita, location at R1, R2, R3, and R4. Additionally, Ethiopia adjusted for YL child's birth order and language of test administration; India adjusted for language of school instruction; and Peru adjusted for language of test administration. cHAZ, conditional height-for-age z-score; EGRA, Early Grades Reading Assessment; HAZ, height-for-age z-score; PPVT, Peabody Picture Vocabulary Test. | | | | | | | | | | | | | | | | | |


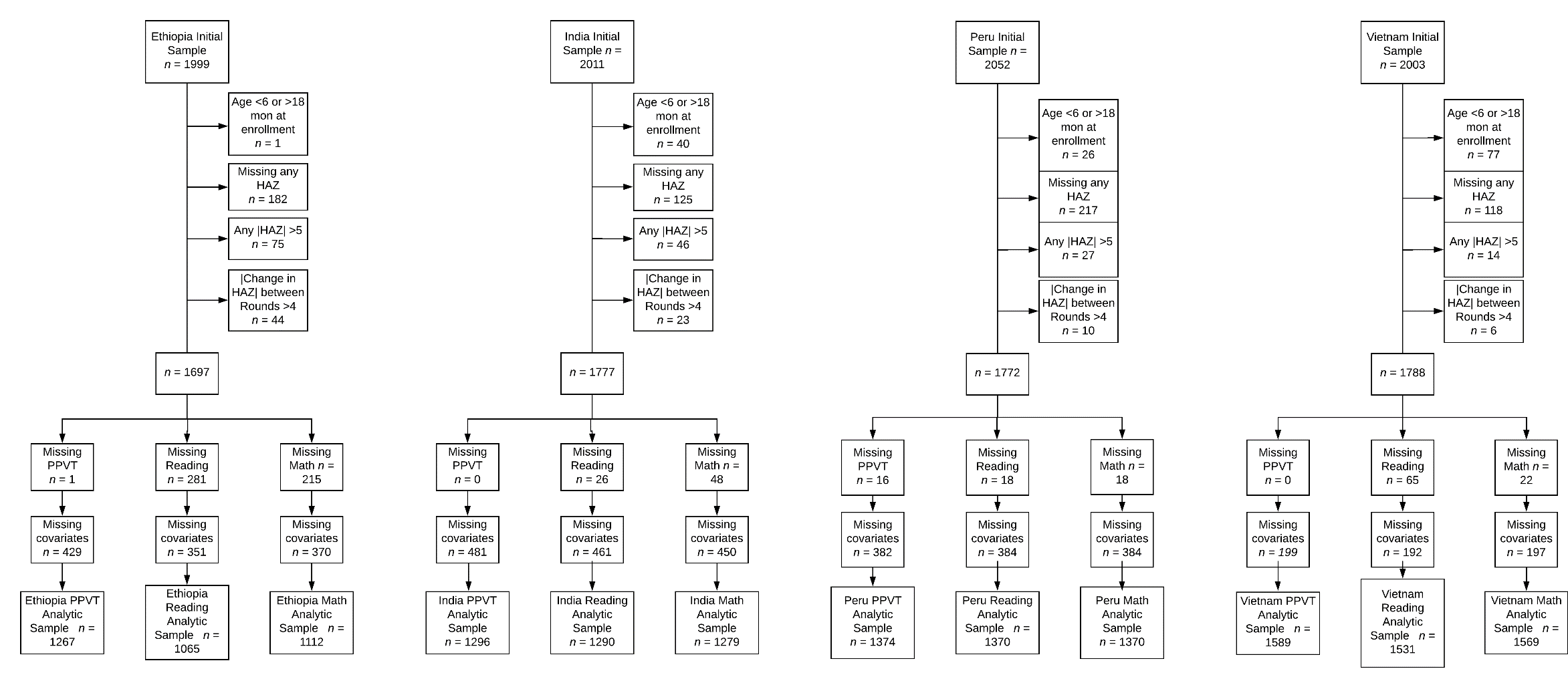
Supplemental Figure 1. Exclusions of children for missing or outlying HAZ, missing achievement test scores, or missing covariate information by country. HAZ, height-for-age z-score.
